# Ion Flux in Roots of Chinese Fir (*Cunninghamia lanceolata* (Lamb.) Hook) under Aluminum Stress

**DOI:** 10.1371/journal.pone.0156832

**Published:** 2016-06-06

**Authors:** Zhihui Ma, Binlong Huang, Shanshan Xu, Yu Chen, Guangqiu Cao, Guochang Ding, Sizu Lin

**Affiliations:** 1 College of Life Sciences, Fujian Agriculture and Forestry University, Fuzhou, Fujian, China; 2 State Forestry Administration Engineering Research Center of Chinese Fir, Fuzhou, Fujian, China; 3 Fujian Provincial Forestry Survey and Design Institute, Fuzhou, Fujian, China; 4 College of Forestry, Fujian Agricultural and Forestry University, Fuzhou, Fujian, China; University of Tasmania, AUSTRALIA

## Abstract

Chinese fir is a tall, fast-growing species that is unique to southern China. In Chinese fir plantations, successive plantings have led to a decline in soil fertility, and aluminum toxicity is thought to be one of the main reasons for this decline. In this study, Non-invasive Micro-test Technology was used to study the effect of aluminum stress on the absorption of 4 different ions in the roots of the Chinese fir clone FS01. The results are as follows: with increased aluminum concentration and longer periods of aluminum stress, the H^+^ ion flow gradually changed from influx into efflux; there was a large variation in the K^+^ efflux, which gradually decreased with increasing duration of aluminum stress; and 1 h of aluminum stress uniformly resulted in Ca^2+^ influx, but it changed from influx to efflux after a longer period of aluminum stress. Changes in the different concentrations of aluminum had the largest influence on Mg^2+^.

## Introduction

Aluminum is the third most abundant element in the earth’s crust, representing approximately 8% of its mass. Ulrich noted that in acidic soils, aluminum toxicity may be one of the primary abiotic stress factors contributing to forest decline [1]. Many studies have demonstrated that toxic aluminum concentrations rapidly inhibit root elongation, with the root tip the major site of aluminum-induced injury, thus resulting in a poor uptake of water and nutrients [2]. The root transition zone in particular and the root apex in general have been identified as critical sites for sensing Al^3+^ toxicity and tolerance to Al^3+^ [3]. Rengel has noted that the effects of aluminum toxicity on the shoots, such as growth inhibition, become evident only after root growth is inhibited by exposure to toxic aluminum levels in the rhizosphere, resulting in mineral nutrition deficiencies in the aboveground tissue [4].

Current knowledge suggests that the detrimental effect of aluminum on plants has several facets, including the competitive inhibition of Mg^2+^ and Ca^2+^ absorption sites on the cell membrane, thereby inhibiting the absorption and transportation of water and ions [5]. Bose has reported that in *Arabidopsis* treated with low pH and aluminum, the aluminum-resistant genotypes accumulated more Mg^2+^, had greater Mg^2+^ influx and had higher intracellular Mg^2+^ concentrations than the aluminum-sensitive genotypes, demonstrating that increased Mg^2+^ uptake correlates with an enhanced capacity of *Arabidopsis* to cope with low pH and combined low pH and aluminum stress [6]. Ca^2+^ is needed for the secretory functions of the cap cells, and aluminum is known to affect cellular Ca^2+^ homeostasis, resulting in a reduction in mucilage secretion [7, 8]. Ryan has shown that aluminum toxicity can inhibit the absorption of Ca^2+^ in the roots [9]. Olivetti has reported that in aluminum-tolerant snapbean, aluminum causes a depolarization of the electrical potential in the root cap cells, possibly due to reduced K^+^ channel conductance [10]. Liu and Luan have reported that aluminum enters plant cells through a Ca^2+^ channel-like pathway and inhibits the K^+^ in the cell by blocking the channels on the cytoplasmic side of the plasma membrane [11]. Nichol has studied the effects of aluminum on the influx of calcium, potassium, and ammonium cations and of nitrate and phosphate anions in an aluminum-sensitive cultivar of barley (*Hordeum vulgare* L.) and found that 100 μmol·L^-1^ aluminum inhibited the influx of Ca^2+^ and K^+^ cations by 69% and 13%, respectively [12].

Although the physiological mechanisms by which aluminum interferes with ion influx have been relatively well studied over the past two decades, such physiological mechanisms in Chinese fir are poorly understood, especially in terms of ion influx levels.

Because of its desirable attributes, which include fast growth, good material, strong wood, hardiness, versatile use and high timber yield per unit, Chinese fir (*Cunninghamia lanceolata*) is one of the most important coniferous evergreen timber tree species in southern China. This species is a major industrial and commercial wood source and is the dominant tree species in China. Currently, Chinese fir that is grown in China is affected by aluminum toxicity, particularly in the south. To alleviate this aluminum toxicity and improve production in acidic soils, it is necessary to understand the relationship between aluminum stress and the uptake of H^+^, K^+^, Ca^2+^ and Mg^2+^ ions in the roots. Hence, our goal in this study was to elucidate the relationship between aluminum stress and the absorption of H^+^, K^+^, Ca^2+^ and Mg^2+^ ions in Chinese fir roots by using Non-invasive Micro-test Technology (NMT; BIO-001A, Younger USA Sci. & Tech. Corp., Amherst, MA). We found that different aluminum concentrations and different durations of aluminum stress treatment affected the absorption of H^+^, K^+^, Ca^2+^ and Mg^2+^ ions in the roots of a Chinese fir clone. A 1-h treatment with increasing concentrations of aluminum had an increasing influence on the absorption of H^+^, K^+^ and Mg^2+^ but not as great an effect on the absorption of Ca^2+^ ions. Under these conditions, the H^+^ ion flow changed gradually from influx to efflux, the K^+^ ion flow was consistently out of the root and showed a high variation in amplitude, and the Ca^2+^ ion flow was primarily into the roots after 1 h of aluminum stress but changed from influx to efflux after a longer treatment. In another set of treatments that lasted for 32 h, increasing concentrations of aluminum had the largest influence of the absorption of H^+^, Ca^2+^ and Mg^2+^ and a weaker effect on the absorption of K^+^ ions. Under these conditions, the H^+^ ion flow changed gradually from influx to efflux, the K^+^ ion flow changed from efflux to weak influx, the Ca^2+^ ion flow changed from influx to an efflux whose velocity decreased after 32 h of aluminum stress, and the Mg^2+^ ion flow was consistently out of the roots and showed a large variation in velocity. In general, our results demonstrated that with increasing aluminum concentrations and increasing duration of stress, the H^+^ ion flow in the roots of Chinese fir gradually changed from influx to efflux, the K^+^ efflux varied greatly and gradually decreased with increasing time under stress, and the Ca^2+^ ion flow was into the roots after 1 h of aluminum stress but changed from influx to efflux after longer periods of stress. Different concentrations of aluminum had the largest influence on Mg^2+^.

## Results

### The effect of aluminum toxicity on root elongation of Chinese fir

**Fig 1:**
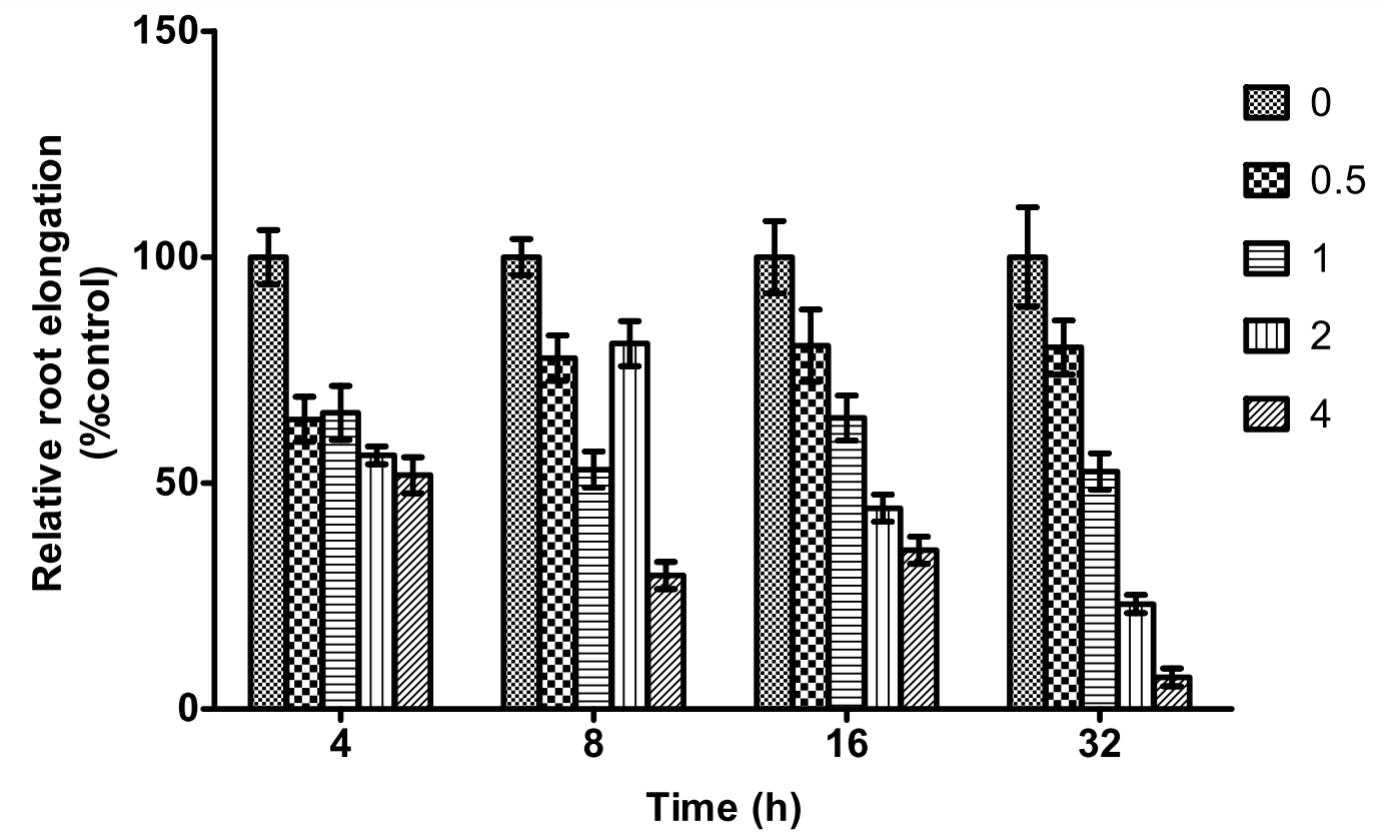
Effect of aluminum toxicity on the relative root elongation of Chinese fir.

We used the seedlings germinated from seeds of the Chinese fir clone NO.40 to evaluate the effect of aluminum toxicity on the root elongation of Chinese fir. Because the root elongation of Chinese fir is not obvious after 1 h of stress with various concentrations of aluminum, we selected 4 h as the first time point for discussing the root elongation of Chinese fir seedlings under aluminum toxicity. Fig 1 shows that the relative root elongation of Chinese fir seedlings shows some differences after 4 h of stress under different aluminum concentrations, but the degree of relative root elongation is greater than 50% in almost all cases. The differences in relative root elongation are especially obvious under stress with various concentrations of aluminum for 16 h and 32 h. For example, the relative root elongation is less than 50% while under 2 mmol·L^-1^ and 4 mmol·L^-1^ AlCl_3_ stress for 16h and is more obviously impaired while under various levels of AlCl_3_ stress for 32 h.

### The structure of the Chinese fir root

**Fig 2:**
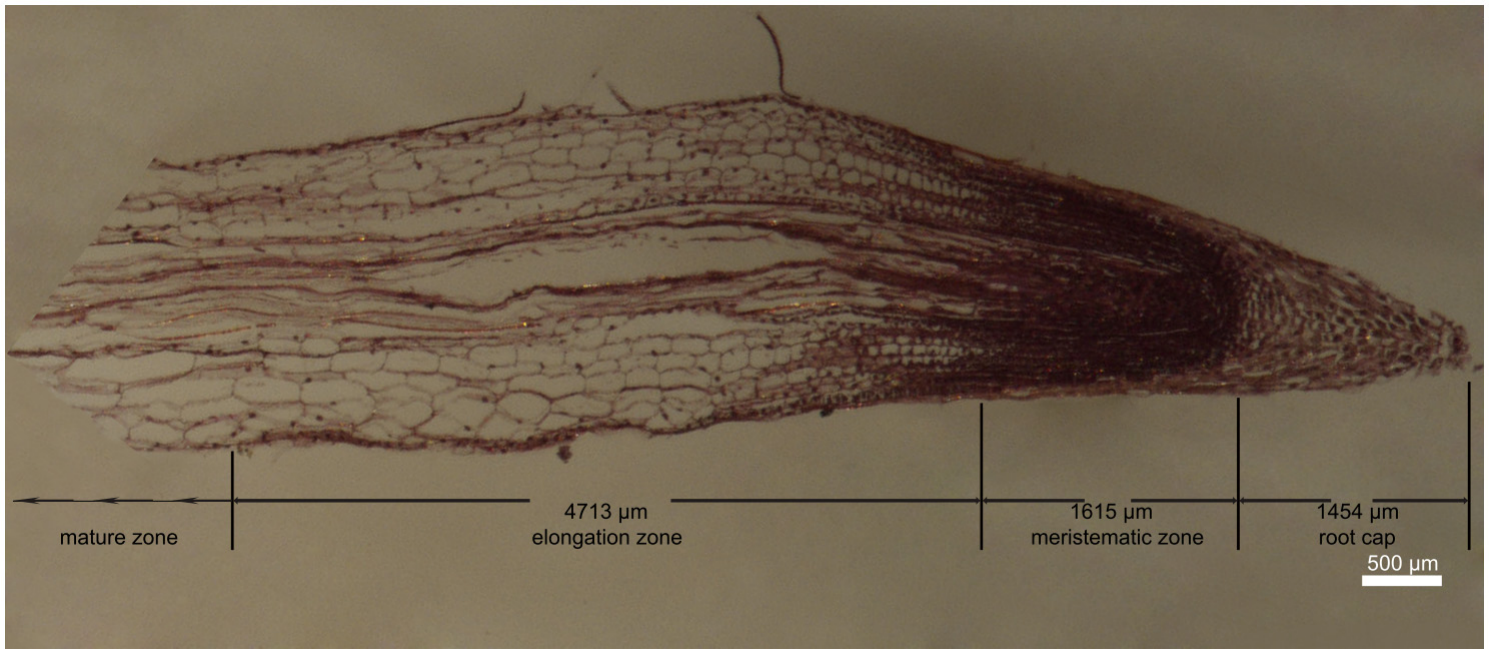
The structure of the Chinese fir root.

The structure of the Chinese fir root was visualized using the conventional method of serial paraffin section. From the paraffin section of the root, we can see that the root tip of Chinese fir has four distinct zones along the longitudinal axis (Fig 2): root cap (zone at the forefront of the root tip that can protect the meristematic zone; approximately 1454 μm long), meristematic zone (region of cell division; approximately 1615μm long), elongation zone (approximately 4713μm long) and mature zone (zone of root hairs).

### Determination of the root tip position for the ion flux measurement

In order to identify an appropriate point in the root tip of Chinese fir for the measurement of ion flux, at the beginning of the study, we chose the positions 0, 200, 500, 800, 1200, 1600, 2000, 2600, 3200, 3800, 4400 and 5000 μm from the root tip for scanning the H^+^ flux measurement. After analyzing the scanning data, we chose the position of 2600 μm from the root tip for the subsequent four sets of measurement of ion flux (Data not shown, see the S1 Fig). From Fig 2, we can see that the five positions 0, 200, 500, 800, and 1200 from the root tip of Chinese fir belonged to root cap, the three positions 1600, 2000, 2600 from the root tip of Chinese fir belonged to meristematic zone, and the other three positions belonged to the root elongation zone.

Real-time flux measurements at 2600 μm are shown in the Supporting Information files (S1–S4 Tables).

### The effects of aluminum stress on the H^+^ flux in Chinese fir roots

**Fig 3:**
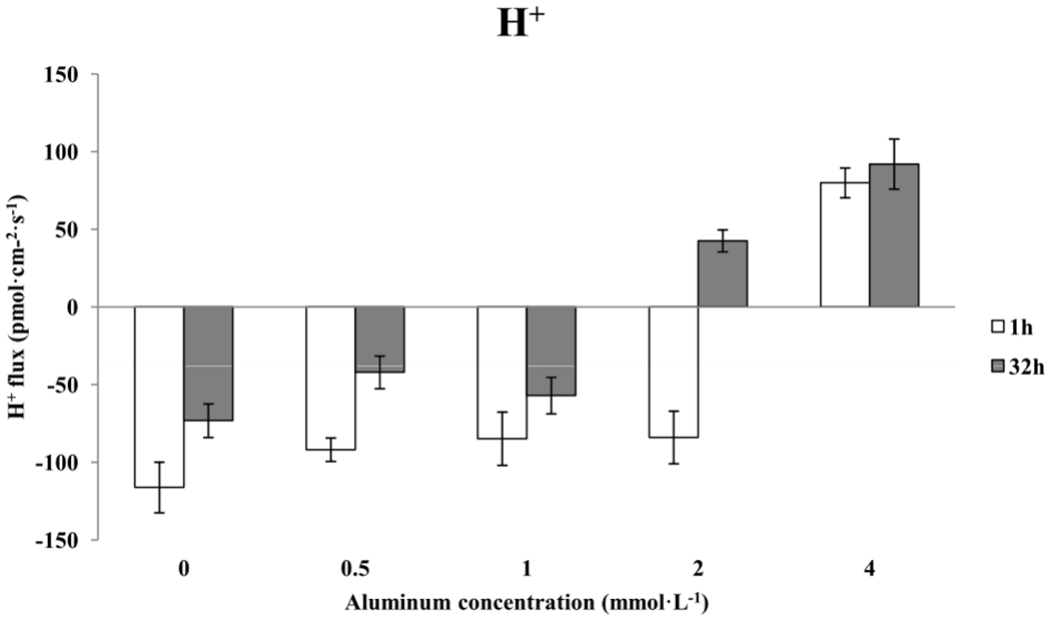
The effects of aluminum stress on H^+^ flux in Chinese fir roots.

After a 1-h treatment with increasing concentrations of aluminum, the influx of H^+^ in the roots of Chinese fir decreased relative to the control group (0 mmol·L^-1^ AlCl_3_), the H^+^ ion flow changed to efflux, and its velocity changed significantly (Fig 3). Unlike in the low-concentration aluminum stress treatments, plants treated with 4 mmol·L^-1^ AlCl_3_ lost H^+^, and the average velocity of H^+^ efflux was approximately 80 pmol·cm^-2^·s^-1^. After 32 h of treatment, the H^+^ flux also changed from influx to efflux. The trend was similar to that for the 1-h treatment, but after 32 h, the H^+^ flux changed from influx to efflux in the 2 mmol·L^-1^ AlCl_3_ treatment (Fig 3). Compared with the control (0 mmol·L^-1^ AlCl_3_), after 32 h, the influx of H^+^ decreased in the presence of 0.5 mmol·L^-1^ AlCl_3_, and the average velocity of H^+^ influx was approximately 42 pmol·cm^-2^·s^-1^. The H^+^ influx decreased in the presence of 1 mmol·L^-1^ AlCl_3_ as well, but it was greater than that of the plants treated with 0.5 mmol·L^-1^ aluminum, and the average velocity of H^+^ influx was approximately 57 pmol·cm^-2^·s^-1^. When the aluminum concentration increased to 2 mmol·L^-1^, the H^+^ flux changed from influx to efflux, and the average velocity of H^+^ efflux was approximately 42 pmol·cm^-2^·s^-1^. In the presence of 4 mmol·L^-1^ aluminum, the H^+^ efflux clearly increased, and its average velocity was approximately 92 pmol·cm^-2^·s^-1^.

### The effects of aluminum stress on the K^+^ flux in Chinese fir roots

**Fig 4:**
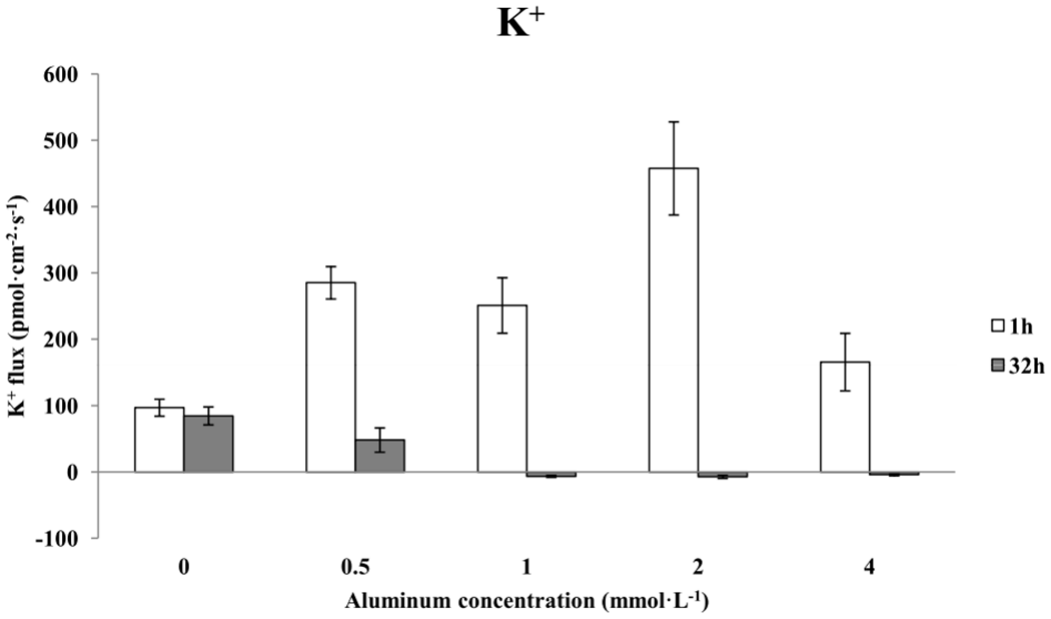
The effects of aluminum stress on K^+^ flux in Chinese fir roots.

A 1-h treatment with increasing concentrations of aluminum consistently resulted in K^+^ efflux from the roots of Chinese fir, and the K^+^ efflux velocity clearly differed from that of the control (0 mmol·L^-1^ AlCl_3_) (Fig 4). In roots treated with 0.5 mmol·L^-1^ AlCl_3_ for 1 h, the average K^+^ efflux velocity was 285 pmol·cm^-2^·s^-1^, which represented an increase of 193.81% over the control. The average K^+^ efflux after 1 h of treatment with 1 mmol·L^-1^ AlCl_3_ was 251 pmol·cm^-2^·s^-1^; this velocity was 158.76% that of the control and 11.93% less than that under the 0.5 mmol·L^-1^ AlCl_3_ treatment. The average K^+^ efflux after 1 h of treatment with 2 mmol·L^-1^ AlCl_3_ was 458 pmol·cm^-2^·s^-1^, which was 372.16% that of the control and was the largest change caused by any aluminum concentration. The velocity of K^+^ efflux fell slightly after 1 h of treatment with 4 mmol·L^-1^ AlCl_3_; the average velocity was approximately 167 pmol·cm^-2^·s^-1^, which was 72.16% faster than in the control. By contrast, after 32 h of treatment with different concentrations of aluminum, the K^+^ flux in the Chinese fir roots gradually changed from efflux to influx. Generally, the K^+^ flux exhibited a declining trend with minimal variation. The average K^+^ efflux after 32 h of treatment with 0.5 mmol·L^-1^ AlCl_3_ was 193.81% less than that of the control. A 32-h treatment with 1 mmol·L^-1^, 2 mmol·L^-1^ or 4 mmol·L^-1^ AlCl_3_ led to K^+^ influx that did not vary substantially; the average influx for each treatment was 7 pmol·cm^-2^·s^-1^, 7 pmol·cm^-2^·s^-1^ and 4 pmol·cm^-2^·s^-1^, respectively.

### The effects of aluminum stress on Ca^2+^ flux in Chinese fir roots

**Fig 5:**
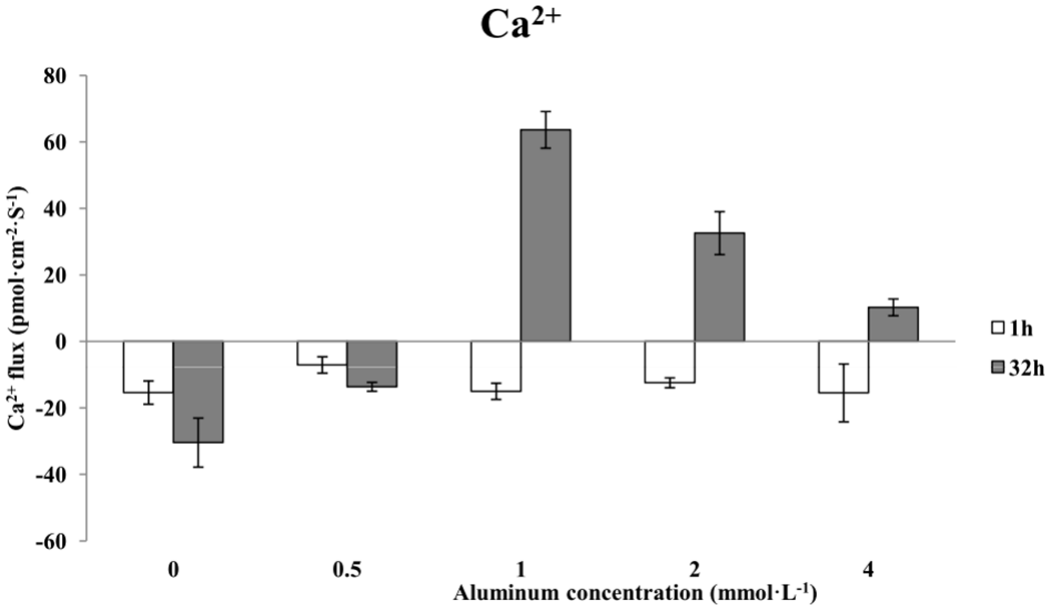
The effects of aluminum stress on Ca^2+^ flux in Chinese fir roots.

Treatment with various concentrations of aluminum for 1 h uniformly resulted in Ca^2+^ influx. The Ca^2+^ influx velocity appeared to decrease slightly but fluctuated at approximately the velocity of the control group (0 mmol·L^-1^ AlCl_3_) (Fig 5). The average Ca^2+^ influx after a 1-h treatment with 0.5 mmol·L^-1^ AlCl_3_ decreased slightly to 6 pmol·cm^-2^·s^-1^, which was 53.33% that of the control. After a 1-h treatment with 1 mmol·L^-1^ AlCl_3_, the average Ca^2+^ influx increased slightly to approximately 15 pmol·cm^-2^·s^-1^. The 2 mmol·L^-1^ AlCl_3_ treatment reduced the average Ca^2+^ influx to 12 pmol·cm^-2^·s^-1^, which was 20.00% less than that of the control. The 1-h treatment with 4 mmol·L^-1^ AlCl_3_ resulted in a slight increase in the average Ca^2+^ influx; the average velocity was approximately 15 pmol·cm^-2^·s^-1^. By contrast, treatment with different concentrations of aluminum for 32 h resulted in a gradual change in the Ca^2+^ flux from influx to efflux, with very clear changes in the average flux velocity (Fig 5). Treatment with 0.5 mmol·L^-1^ AlCl_3_ led to Ca^2+^ influx with a velocity of 14 pmol·cm^-2^·s^-1^, which was 53.33% less than that of the control. Treatment with 1 mmol·L^-1^ AlCl_3_ caused a change in the Ca^2+^ flux from influx to efflux, with an average velocity of approximately 64 pmol·cm^-2^·s^-1^. When the aluminum concentration increased to 2 mmol·L^-1^, the Ca^2+^ efflux was dramatically reduced and the average velocity was approximately 33 pmol·cm^-2^·s^-1^. When the aluminum concentration increased to 4 mmol·L^-1^, the Ca^2+^ efflux was further reduced, with an average velocity of 10 pmol·cm^-2^·s^-1^.

A comparison between treatments with the same aluminum concentration for different lengths of time showed that the direction of Ca^2+^ flux in plants treated with 0.5 mmol·L^-1^ AlCl_3_ was similar to that of the control and that there was greater Ca^2+^ influx after a 1-h treatment with 0.5 mmol·L^-1^ AlCl_3_ than with a 32-h treatment. As the aluminum concentration increased to 1 mmol·L^-1^, the Ca^2+^ flux changed from influx after 1 h to efflux after 32 h. The 1- and 32-h treatments with 2 mmol·L^-1^ or 4 mmol·L^-1^ aluminum caused changes in Ca^2+^ flux that were similar to those observed in the group treated with 1 mmol·L^-1^ AlCl_3_: the 1-h treatment resulted in Ca^2+^ influx, and the 32-h treatment resulted in Ca^2+^ efflux, but the overall Ca^2+^ flux was significantly greater after the 32-h treatment.

### The effects of aluminum stress on Mg^2+^ flux in Chinese fir roots

**Fig 6:**
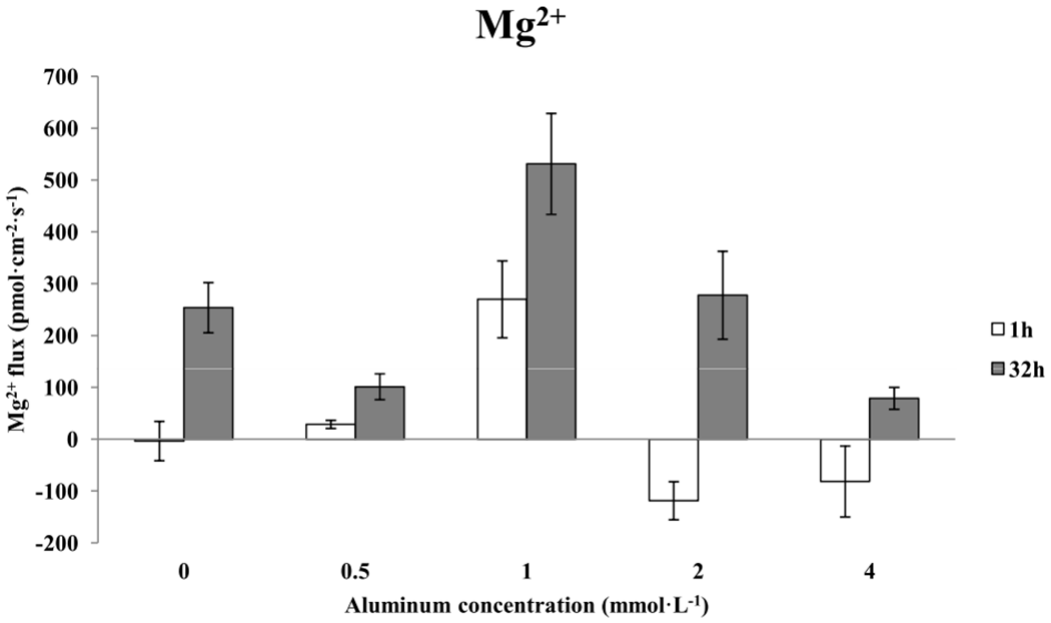
The effects of aluminum stress on Mg^2+^ flux in Chinese fir roots.

Treating Chinese fir with increasing concentrations of aluminum for 1 h caused the Mg^2+^ flux in the roots to change from influx to efflux and then back to influx (Fig 6). The average Mg^2+^ efflux after a 1-h treatment with 0.5 mmol·L^-1^ AlCl_3_ was 29 pmol·cm^-2^·s^-1^. The average Mg^2+^ efflux after treatment with 1 mmol·L^-1^ AlCl_3_ increased dramatically to approximately 270 pmol·cm^-2^·s^-1^. The Mg^2+^ flux after treatment with 2 mmol·L^-1^ AlCl_3_ changed from efflux to influx, with an average influx of 119 pmol·cm^-2^·s^-1^. As the aluminum concentration increased to 4 mmol·L^-1^, the Mg^2+^ influx decreased slightly; the average velocity of Mg^2+^ influx was approximately 82 pmol·cm^-2^·s^-1^. By contrast, 32-h treatments with different concentrations of aluminum uniformly resulted in Mg^2+^ efflux, whose velocity clearly varied (Fig 6). The average Mg^2+^ efflux after a 32-h treatment with 0.5 mmol·L^-1^ AlCl_3_ decreased to 14 pmol·cm^-2^·s^-1^, which was 60.24% less than that of the control. The average Mg^2+^ efflux after a 32-h treatment with 1 mmol·L^-1^ AlCl_3_ dramatically increased to 531 pmol·cm^-2^·s^-1^, which represented an increase of 109.05% over the control. When the aluminum concentration was increased to 2 mmol·L^-1^, the average Mg^2+^ efflux decreased to approximately 278 pmol·cm^-2^·s^-1^, and when the aluminum concentration increased to 4 mmol·L^-1^, the Mg^2+^ efflux further decreased to 79 pmol·cm^-2^·s^-1^.

A comparison of treatments with the same aluminum concentrations for different lengths of time showed that the changes in Mg^2+^ flux caused by the 0.5 mmol·L^-1^ AlCl_3_ treatment were similar to those observed with 1 mmol·L^-1^ AlCl_3_; the velocity of the Mg^2+^ flux after 1 h was greater than that after 32 h. The Mg^2+^ flux in the roots after treatment with 2 mmol·L^-1^ AlCl_3_ was similar to that after treatment with 4 mmol·L^-1^ AlCl_3_; there was an influx of Mg^2+^ after a 1-h treatment and an efflux after 32 h. When the aluminum concentration was increased from 2 mmol·L^-1^ to 4 mmol·L^-1^, the average velocity of the Mg^2+^ flux gradually decreased across the 1-h treatments, and the change in the Mg^2+^ efflux was similar.

## Discussion

Various studies in plants have shown that clear symptoms of aluminum toxicity first appear in the roots and are characterized by a significant inhibition of root elongation, interference in the absorption of water and nutrients, increased permeability of the cell membrane, and the inhibition of the transmembrane transport of Ca^2+^ and Mg^2+^, resulting in a drastic drop in the net absorption rates of Ca^2+^, Mg^2+^ and other nutrients, with a particular decrease in Ca^2+^ influx [13–24]. Foy has reported that aluminum depresses the uptake of Mg and Ca, while the effects of aluminum stress on K uptake depend on the growth conditions [25]. The accumulation of aluminum in the cell walls of the root tip is a precondition for aluminum toxicity in plants and is an important characteristic of aluminum-sensitive plants [26–28]. Therefore, studying the effect of aluminum stress on ion absorption and ion flow in the roots of a Chinese fir clone can provide a theoretical basis and a reference for understanding the response mechanism of Chinese fir clones in resistance to aluminum toxicity. This study showed that varying concentrations of aluminum, as well as aluminum stress of varying durations (1 h or 32 h), affected the absorption and ion flow of H^+^, K^+^, Ca^2+^ and Mg^2+^ in the roots of Chinese fir clones. We chose 1 h and 32 h as the aluminum stress of varying durations for the ion flux measurement based on an experiment by using germinated seedlings to evaluate the effect of aluminum toxicity on the root elongation. In that experiment we tested four concentrations of aluminum (0.5, 1, 2 and 4 mmol·m^-1^) and a control condition to investigate the effects of different aluminum concentrations on the root elongation of Chinese fir under aluminum stress for 1 h, 4 h, 8 h, 16 h and 32 h. We found that the relative root elongation was clearly inhibited after aluminum stress for 16 h and 32 h, especially at 32 h (Data not shown). Therefore, we chose 1 h and 32 h as the key time points of aluminum stress for discussing the effects of different concentrations of aluminum on the measurement of ion flux in Chinese fir root.

In our experiment we found that the H^+^ influx into the roots of Chinese fir gradually decreased and ultimately changed to efflux with increasing concentrations of aluminum. The ion flow velocity changed significantly after a 1-h treatment with different aluminum concentrations, whereas the H^+^ flux gradually changed from influx to efflux after a 32-h treatment with different aluminum concentrations. Different concentrations of aluminum and different durations of aluminum stress can either inhibit or promote plasma membrane H^+^-ATPase activity, and this finding is consistent with the results reported by Façanha [29] and Shen [30]. Kasai found that aluminum stress can increase K^+^ efflux and the activity of ATP- and PPi-dependent H^+^ pumps in the tonoplast-enriched membrane vesicles of barley roots [31]. Matsumoto reported that aluminum can induce vacuole membrane H^+^ pump activity and hypothesized that this phenomenon is a plant adaptation; increases in H^+^ transport can maintain the intracellular pH balance, and Al^3+^ can be compartmentalized into the vacuole, ultimately reducing the aluminum toxicity [32]. Therefore, the effect of vacuoles in plant resistance to metal ions has been confirmed, and they may also play an important role in plant resistance to aluminum toxicity. Wang suggested that the destruction of molecular structures is a major cause of cellular damage and that the vacuolar sequestration of Al^3+^ is an important aluminum resistance mechanism in rice [33].

Potassium is a most important plant nutrient. It not only promotes respiration and the synthesis of nucleic acids and proteins but also improves the quality and stress tolerance of plants in addition to its role in turgor-driven movements, osmoregulation and maintenance of the plasma membrane potential [2, 9, 34]. As K^+^ is required for plant growth, the plant roots must take up sufficient amounts of K^+^ from the soil solution or rhizospheric environment and transport the nutrient to the aerial parts of plant [34]. In our research, we found that increasing concentrations of aluminum uniformly caused K^+^ efflux in the roots of Chinese fir, and the velocity of this efflux clearly differed from that of the control. In contrast, after 32 h of stress with different concentrations of aluminum, the K^+^ flux in the Chinese fir roots gradually changed from efflux to influx, and generally, the rate of flux exhibited a declining trend with minimal variation. This effect may be due to the aluminum’s inhibition of the K^+^ channels in the root hair zone and in the guard cells. To some extent, this result was similar to that of Liu, who showed that aluminum inhibited K^+^ absorption in plants by blocking the cytoplasmic side of the K^+^ channels [11]. Future studies should investigate the effect of aluminum stress on K^+^ absorption in the roots of Chinese fir clones by using a K^+^ inhibitor to block the K^+^ channels.

In the present study, we found that a 1-h treatment with increasing concentrations of aluminum uniformly resulted in Ca^2+^ influx into the roots of Chinese fir. By contrast, after 32 h of aluminum stress, the Ca^2+^ flux gradually changed from influx to efflux in response to different aluminum concentrations, and the Ca^2+^ flux velocity clearly changed. This result may be due to Al^3+^ competing for the receptor binding sites of special membrane channels, thereby hindering Ca^2+^ membrane binding, affecting the activity of GTP-dependent Ca^2+^ transmembrane transport proteins, and causing a gradual decrease in Ca^2+^ influx that eventually leads to Ca^2+^ efflux [35]. After 32 h of aluminum stress with increasing concentrations of aluminum, the flux of Ca^2+^ in the roots of Chinese fir temporarily increases. This result is consistent with that reported by Ma, who found that aluminum stress disrupted the Ca^2+^ balance in the root tip cells of rye, resulting in a temporary rise in Ca^2+^ [36]. Another report showed that aluminum may prevent plants from absorbing Ca^2+^, and aluminum toxicity always results in a lack of calcium in the plant and increased Ca^2+^ efflux from the roots. Our results were consistent with these reports to a certain extent. Future studies should examine the effect of calcium on alleviating aluminum toxicity in Chinese fir clones by adding exogenous calcium.

Magnesium is an essential nutrient for plant growth. It is an essential constituent of the chlorophyll molecule and an activator of various enzymes in plant respiration and photosynthesis. However, the free Mg^2+^ level in the cytosol is strictly regulated due to its role in membrane ionic currents [37, 38]. In the present study, we found that after 1 h of stress with increasing concentrations of aluminum, the Mg^2+^ flux in the roots of Chinese fir changed from influx to efflux and then back to influx. By contrast, 32 h of aluminum stress uniformly resulted in Mg^2+^ efflux, and the velocity of the Mg^2+^ flux clearly varied. To some extent, these results were similar to those of previous studies that reported that aluminum can inhibit Mg^2+^ absorption in plant roots [16, 39]. Bose has reported that the Mg^2+^ content in the vacuole of *Arabidopsis* increased after 7 d under low pH and aluminum stress and that the root absorption of Mg^2+^ increased under short-duration aluminum stress [6]. Some researchers have reported that a number of transporter proteins have been identified which are involved in the transport of Mg^2+^ across biological membranes. Whether there is a similar transport mechanism of Mg^2+^ in Chinese fir root under aluminum stress is still unknown. Efforts are underway to address this question.

## Conclusion

We chose the position 2600 μm from the root tip of Chinese fir, belonging to the meristematic zone, as the NMT measurement point. We found that aluminum stress at different concentrations and for different lengths of time had different effects on the absorption of H^+^, K^+^, Ca^2+^ and Mg^2+^ ion flow in the roots of a Chinese fir clone. With increasing concentrations of aluminum stress, the greatest influence was observed on the absorption of H^+^, K^+^ and Mg^2+^ ion flow in the root of the studied Chinese fir clone with various concentrations of aluminum stresses for over 1 h, and there was less of an effect on the absorption of Ca^2+^ ion flow. In this case, the H^+^ ion flow in the root of the clone changed gradually from influx to efflux; the K^+^ ion flow in the root of the clone was all efflux with high amplitude variation; Ca^2+^ ion flow in the root of the clone was all influx after 1 h of aluminum stress, and it changed from influx to larger efflux under prolonged aluminum stress. With the increase in aluminum stress concentration, there was a strongly marked effect on the absorption of H^+^, Ca^2+^ and Mg^2+^ ion flow in the root of the clone after different aluminum concentration stresses over a period of 32 h, and there was less effect on the absorption of K^+^ ion flow; in this case, the H^+^ ion flow changed gradually from influx to efflux; the K^+^ ion flow changed from efflux to a small influx; Ca^2+^ ion flow changed from influx to efflux and the efflux velocity decreased after aluminum stress for 32 h; and Mg^2+^ ion flow in the root of Chinese fir clone was all efflux with a larger variation of efflux velocity. In general, with increased aluminum stress concentration and the extension of aluminum stress time, the absorption of H^+^ ion flow changed gradually from influx to efflux; there was substantial variation in the K^+^ efflux, and the K^+^ efflux changed gradually under long-duration aluminum stress; Ca^2+^ ion flow was all influx after an aluminum stress of 1 h, and it changed from influx to larger efflux under long-duration aluminum stress. The strongest influence observed involved the effects of different concentration of aluminum stress on Mg^2+^.

## Materials and Methods

### Plant materials and growth conditions

The effects of aluminum toxicity on the root growth of Chinese fir were evaluated by using three-generation pedigree seeds of Chinese fir clone NO. 40, which were provided by the Fujian Youxi State-Owned Forest, and the seeds were collected on November 20, 2011. The seeds of Chinese fir clone NO. 40 were soaked in water with an initial temperature of 45°C for 24 h, after which the seeds were disinfected with 0.5% potassium permanganate for 20 minutes. Then, the seed-germination experiment was carried out on filter paper via the vertical glass-plate sprout method. The entire seed-germination experiment was carried out in a controlled growth chamber at 25°C±3°C, with 70±2% constant relative humidity and light intensity of 2000 lux (photoperiod 12 h:12 h (8:00–20:00)). After 7 days, the germinated seedlings showing consistent growth were used to evaluate the effect of aluminum toxicity on the root elongation of Chinese fir seedlings.

The Chinese fir seedlings were provided by the State Forestry Administration Engineering Research Center for Chinese Fir. This study did not involve endangered or protected species, and the materials belonged to the State Forestry Administration Engineering Research Center of Chinese Fir, China.). Short (2 cm) shoots were initially grown in basic MS (Murashige and Skoog) medium, pH 5.8, containing 0.25 mg·L^-1^ IBA and 0.33 mg·L^-1^ 6-BA and supplemented with 4% sucrose and 0.6% agar. After 60 d, the short, 4-cm shoots were shifted to a rooting medium for plantlet regeneration (1/4 MS medium, pH 5.4, containing 0.14 mg·L^-1^ IBA and 0.075 mg·L^-1^ NAA and supplemented with 2% sucrose and 0.65% carrageenan), placed in the dark for 7 d at 25±3°C, and then transferred to the light at 25±3°C. The photoperiod was 12 h:12 h (8:00–20:00), and the light intensity was 2000 lux.

### Evaluation of root growth of Chinese fir under aluminum toxicity

We used germinated seedlings of the Chinese fir clone NO. 40 to evaluate the effect of aluminum toxicity on the root elongation of Chinese fir. In this experiment, we designed five treatments that included one control group (0.5 mmol·L^-1^ CaCl_2_ without AlCl_3_) and four aluminum-treated groups (0.5 mmol·L^-1^ CaCl_2_+0, 0.5, 1, 2 or 4 mmol·L^-1^ AlCl_3_) with pH 4.0, each treatment consisting of 24 7-day-old seedlings of Chinese fir, and including 3 independent biological repetitions. After aluminum stress for 4 h, 8 h, 16 h and 32 h, the root length was measured using Adobe Photoshop CS5 (Adobe Systems, CA, USA). The relative root elongation was calculated as the ratio of root elongation with aluminum toxicity to root elongation without aluminum toxicity.

### Paraffin section of Chinese fir root

The structure of Chinese fir root was visualized using the conventional method of serial paraffin section with some modifications. First, 1 cm root segments (from the tip) of 5-day-old germinated seedling were excised and immediately placed in FAA fixing solution (90 ml 50% ethanol+5 ml formaldehyde+5 ml acetic acid) for 24 h at room temperature. After fixation, the root tissues were processed as follows: rinsing (3 rinses in 50% ethanol, each time no less than 20 min), dehydrated (70% ethanol, 1 h; 80% ethanol, 1 h; 90% ethanol, 1 h; 95% ethanol, 1 h; 100% ethanol, 1 h twice), made transparent (1/2 ethanol:1/2 xylene, 1 h; xylene, 20 min; xylene, 5 min), waxed (1/2 xylene:1/2 paraffin, 6 h at 37°C; paraffin, 1 h and paraffin, 1 h at 60°C), embedded in paraffin, sectioned (10μm), dyed (stained 4–6 h with hematoxylin and eosin (HE) according to standard procedures), rinsed with water for 2 min, dehydrated (50% ethanol, 2 min; 70% ethanol, 2 min; 85% ethanol, 2 min), redyed (1% eosin, 3 min), dehydrated (95% ethanol, 3 min; 100% ethanol, 3 min), made transparent (1/2 ethanol:1/2 xylene, 3 min; xylene, 3 min; xylene, 3 min), and sealed using Permount mounting medium prepared with 3:1 Rhamsan gum:xylene).

### Aluminum treatment for the NMT experiment

After the seedlings had grown for 30 d in the rooting medium, seedlings with similar initial root lengths were washed with distilled water, inserted through a foam support plate and transferred to a plastic container filled with Hoagland-Arnon solution (0.51 g·L^-1^ KNO_3_, 0.136 g·L^-1^ KH_2_PO_4_, 0.49 g·L^-1^ MgSO_4_·7H_2_O, 0.82 g·L^-1^ Ca(NO_3_)_2_·4H_2_O, 0.0139 g·L^-1^ FeSO_4_·7H_2_O, 0.01865 g·L^-1^ EDTA-Na_2_, 2.86 mg·L^-1^ H_3_BO_3_, 0.09304 mg·L^-1^ Na_2_MoO_4_·2H_2_O, 1.81 mg·L^-1^ MnCl_2_·4H_2_O, 0.08 mg·L^-1^ CuSO_4_·5H_2_O, 0.22 mg·L^-1^ ZnSO_4_·7H_2_O, pH 4.0) containing 0, 0.5, 1, 2 or 4 mmol·L^-1^ AlCl_3_. The aluminum stress experiment was performed in a controlled growth chamber at 25°C with a 12 h light:12 h dark photoperiod, 60% constant relative humidity, and a light intensity of 2000 lux during the day. Each treatment was repeated ten times, and each replicate consisted of 80 seedlings. To measure the net flux of H^+^, K^+^, Ca^2+^ and Mg^2+^ in the roots after treatment with aluminum for 1 h and 32 h, 10 seedlings were used in each treatment, and the net flux for each sample was measured for 15 min.

### Steady-state measurements of net H^+^, K^+^, Ca^2+^ and Mg^2+^ flux

The net fluxes of H^+^, K^+^, Ca^2+^ and Mg^2+^ in the Chinese fir roots were measured using Non-invasive Micro-test Technology (NMT, NMT100 Series, Younger USA LLC, Amherst, MA 01002, USA; Xuyue (Beijing) Sci. & Tech. Co., Ltd., Beijing, China).

All electrodes used for steady-state recordings were typically adjusted 2–3 times via calibration throughout the test procedure. The ion flux rate was calculated using Fick’s law of diffusion: J = -DJ (dc/dx), where J is the ion flux in the x direction, dc represents the difference in ion concentration, dx is the microelectrode’s movement between two positions, dc/dx is the ion concentration gradient, and D represents the ion diffusion coefficient in a particular medium.

### Experimental protocols for NMT measurements

Roots were sampled from plants grown in the different concentrations of aluminum, rinsed with distilled water and incubated for 15 min in basic measuring solution (0.1 mmol·L^-1^ CaCl_2_, 0.1 mmol·L^-1^ KCl, 0.1 mmol·L^-1^ MgCl_2_, 0.3 mmol·L^-1^ MES, pH 4.5) for equilibration. Then, the roots were fixed between two filters and small stones, and the H^+^, K^+^, Mg^2+^ and Ca^2+^ fluxes in the roots were measured along the root apex (0–2600 μm from the root tip) using Non-invasive Micro-test Technology (NMT, NMT100 Series, Younger USA LLC, Amherst, MA01002, USA; Xuyue (Beijing) Sci. & Tech. Co., Ltd., Beijing, China). The ion fluxes were measured over a recording period of 15 min. At the beginning, in order to determine the appropriate measuring position of the root tip for the ion flux measurement, we chose the positions 0, 200, 500, 800, 1200, 1600, 2000, 2600, 3200, 3800, 4400 and 5000 μm from the root tip of Chinese fir root under aluminum stress for 1 h or 32 h (aluminum concentrationsof 0, 1, 2 and 4 mmol·L^-1^) for scanning the H^+^ flux measurement. Each plant was measured once, and two plants were measured for each treatment.

### Statistical analysis

All the data were initially processed using Excel software (Excel 2003, Microsoft, Redmond, WA), and the subsequent statistical analysis of the data was conducted with SPSS (Version18.0, SPSS Institute, Chicago, IL, USA). The figures were drawn using GraphPad Prism5.0 (GraphPad Software, Inc., San Diego, CA) or Excel 2007 (Microsoft, Redmond, WA). Images were processed using Adobe Photoshop CS5 and Adobe Illustrator CS5 (Adobe Systems, CA, USA).

## Supporting Information

S1 Fig The H^+^ flux of different distance from the root tip of Chinese fir. (JPG)

S1 Table Real-time flux measurements of H^+^ at 2600 μm in Chinese fir root. (XLSX)

S2 Table Real-time flux measurements of K^+^ at 2600 μm in Chinese fir root. (XLSX)

S3 Table Real-time flux measurements of Ca^2+^ at 2600 μm in Chinese fir root. (XLSX)

S4 Table Real-time flux measurements of Mg^2+^ at 2600 μm in Chinese fir root. (XLSX)

## References

[1] Ulrich B, Mayer R, Khanna P. Chemical changes due to acid precipitation in a Loess-derived soil in Central Europe. Soil Sci. 1980;130(4):193–9.

[2] Ryan PR, Kochian LV. Interaction between aluminum toxicity and calcium uptake at the root apex in near-isogenic lines of wheat (*Triticum aestivum* L.) differing in aluminum tolerance. Plant Physiol. 1993;102(3):975–82. 12231883 10.1104/pp.102.3.975 PMC158871

[3] Chen L, Wang T, Zhao M, Tian Q, Zhang WH. Identification of aluminum-responsive microRNAs in Medicago truncatula by genome-wide high-throughput sequencing. Planta. 2012;235(2):375–86. 10.1007/s00425-011-1514-9 21909758

[4] Rengel Z. Tansley review No. 89. Uptake of aluminium by plant cells. New Phytol. 1996:389–406.

[5] Cao Y, Lou Y, Han Y, Shi J, Wang Y, Wang W, et al Al toxicity leads to enhanced cell division and changed photosynthesis in *Oryza rufipogon* L. Mol Biol Rep. 2011;38(8):4839–46. 10.1007/s11033-010-0618-9 21132530

[6] Bose J, Babourina O, Shabala S, Rengel Z. Low-pH and aluminum resistance in arabidopsis correlates with high cytosolic magnesium content and increased magnesium uptake by plant roots. Plant & cell physiology. 2013;54(7):1093–104. 23620479 10.1093/pcp/pct064

[7] Marschner H. Mineral nutrition of higher plants Mineral nutrition of higher plants. 1995;(Ed. 2).

[8] Kawano T, Kadono T, Furuichi T, Muto S, Lapeyrie F. Aluminum-induced distortion in calcium signaling involving oxidative bursts and channel regulation in tobacco *BY*-2 cells. Biochem Biophys Res Commun. 2003;308(1):35–42. 12890476 10.1016/s0006-291x(03)01286-5

[9] Ryan PR, Kinraide TB, Kochian LV. Al^3+^-Ca^2+^ interactions in aluminum rhizotoxicity. Planta. 1993;192(1):98–103.

[10] Olivetti GP, Cumming JR, Etherton B. Membrane potential depolarization of root cap cells precedes aluminum tolerance in snapbean. Plant Physiol. 1995:123–9.

[11] Liu K, Luan S. Internal aluminum block of plant inward K^+^ channels. The Plant cell. 2001;13(6):1453–65. 11402172 10.1105/tpc.13.6.1453 PMC135577

[12] Nichol BE, Oliveira LA, Glass A, Siddiqi MY. The effects of aluminum on the influx of Calcium, Potassium, Ammonium, Nitrate, and Phosphate in an aluminum-sensitive cultivar of Barley (*Hordeum vulgare* L.). Plant physiol. 1993;101(4):1263–6. 12231781 10.1104/pp.101.4.1263 PMC160648

[13] Zheng SJ, Ma JF, Matsumoto H. High aluminum resistance in buckwheat I. Al-induced specific secretion of oxalic acid from root tips. Plant Physiol. 1998;117(3):745–51. 9662517 10.1104/pp.117.3.745 PMC34929

[14] Zhao X-J, Sucoff E, Stadelmann EJ. Al^3+^ and Ca^2+^ alteration of membrane permeability of Quercus rubra root cortex cells. Plant Physiol. 1987;83(1):159–62. 16665194 10.1104/pp.83.1.159 PMC1056316

[15] Rengel Z, Robinson DL. Competitive Al^3+^ inhibition of net Mg^2+^ uptake by intact Lolium multiflorum roots I. Kinetics. Plant Physiol. 1989;91(4):1407–13. 16667193 10.1104/pp.91.4.1407 PMC1062198

[16] Rengel Z, Robinson D. Aluminum and plant age effects on adsorption of cations in the Donnan free space of ryegrass roots. Plant Soil. 1989;116(2):223–7.

[17] Rengel Z. Competitive Al^3+^ inhibition of net Mg^2+^ uptake by intact *Lolium multiflorum* roots II. Plant age effects. Plant Physiol. 1990;93(3):1261–7. 16667588 10.1104/pp.93.3.1261 PMC1062661

[18] Poschenrieder C, Gunsé B, Corrales I, Barceló J. A glance into aluminum toxicity and resistance in plants. Sci Total Environ. 2008;400(1):356–68. 18657304 10.1016/j.scitotenv.2008.06.003

[19] Matsumoto H. Cell biology of aluminum toxicity and tolerance in higher plants. Int Rev Cytol. 2000;200:1–46. 10965465 10.1016/s0074-7696(00)00001-2

[20] Ma JF, Hiradate S, Matsumoto H. High aluminum resistance in buckwheat II. Oxalic acid detoxifies aluminum internally. Plant Physiol. 1998;117(3):753–9. 9662518 10.1104/pp.117.3.753 PMC34930

[21] Lazof DB, Goldsmith JG, Rufty TW, Linton RW. Rapid uptake of aluminum into cells of intact soybean root tips (a microanalytical study using secondary ion mass spectrometry). Plant Physiol. 1994;106(3):1107–14. 12232392 10.1104/pp.106.3.1107 PMC159637

[22] Kochian LV. Cellular mechanisms of aluminum toxicity and resistance in plants. Annu Rev Plant Biol. 1995;46(1):237–60.

[23] Cumming J, Eckert R, Evans L. Effect of aluminum on potassium uptake by red spruce seedlings. Canadian journal of botany. 1985;63(6):1099–103.

[24] Barceló J, Poschenrieder C. Plant water relations as affected by heavy metal stress: a review. J Plant Nutr. 1990;13(1):1–37.

[25] Foy CD. Plant adaptation to acid, aluminum-toxic soils. Communications in Soil Science & Plant Analysis. 1988;19(7–12):959–87.

[26] Zhang Z, Wang H, Wang X, Bi Y. Nitric oxide enhances aluminum tolerance by affecting cell wall polysaccharides in rice roots. Plant cell reports. 2011;30(9):1701–11. 10.1007/s00299-011-1078-y 21553108

[27] Wang H, Chen RF, Iwashita T, Shen RF, Ma JF. Physiological characterization of aluminum tolerance and accumulation in tartary and wild buckwheat. The New phytologist. 2015;205(1):273–9. 10.1111/nph.13011 25195800

[28] Li J, Liu J, Dong D, Xia X, McCouch SR, Kochian LV, editors. Natural variation in *NRAT*1 alters expression and function of a key gatekeeper for Rice aluminum tolerance. Plant and Animal Genome XXII Conference; 2014: Plant and Animal Genome.

[29] Façanha AR, Okorokova-Façanha AL. Inhibition of phosphate uptake in corn roots by aluminum-fluoride complexes. Plant Physiol. 2002;129(4):1763–72. 12177489 10.1104/pp.001651 PMC166764

[30] Shen H, He LF, Sasaki T, Yamamoto Y, Zheng SJ, Ligaba A, et al Citrate secretion coupled with the modulation of soybean root tip under aluminum stress. Up-regulation of transcription, translation, and threonine-oriented phosphorylation of plasma membrane H^+^-ATPase. Plant Physiol. 2005;138(1):287–96. 15834009 10.1104/pp.104.058065 PMC1104183

[31] Kasai M, Sasaki M, Yamamoto Y, Matsumoto H. Aluminum stress increases K^+^ efflux and activities of ATP-and PPi-dependent H^+^ pumps of tonoplast-enriched membrane vesicles from barley roots. Plant Cell Physiol. 1992;33(7):1035–9.

[32] Matsumoto H. Biochemical mechanism of the toxicity of aluminium and the sequestration of aluminium in plant cells Plant-Soil interactions at low pH: Springer; 1991 p. 825–38.

[33] Wang CY, Shen RF, Wang C, Wang W. Root protein profile changes induced by Al exposure in two rice cultivars differing in Al tolerance. J Proteomics. 2013;78:281–93. 10.1016/j.jprot.2012.09.035 23059537

[34] Mitra GN. Regulation of Nutrient Uptake by Plants: Springer; 2015.

[35] Qing Y, Huabin J. The effect of aluminum stress on N, P and Ca absorption of peanut varieties (in Chinese). Chinese Journal of oil crop sciences. 2000;122(2):68–73.

[36] Q MA, RENGEL Z, KUO J. Aluminium toxicity in rye (Secale cereale): root growth and dynamics of cytoplasmic Ca2+ in intact root tips. Ann Bot. 2002;89(2):241–4. 12099355 10.1093/aob/mcf017 PMC4233785

[37] Li M, Shengchang Y. Impact of Al on accumulations of several metal elements in Brugiera sexangula seedlings (in Chinese). Marine Sciences. 2010;34(8):41–5.

[38] Chen Q, Kan Q, Wang P, Yu W, Yu Y, Zhao Y, et al Phosphorylation and interaction with the 14-3-3 protein of the plasma membrane H^+^-ATPase are involved in the regulation of magnesium-mediated increases in aluminum-induced citrate exudation in Broad Bean (*Vicia faba*. L). Plant Cell Physiol. 2015:pcv038. 10.1093/pcp/pcv038 25745032

[39] Kinraide T. Toxicity factors in acidic forest soils: attempts to evaluate separately the toxic effects of excessive Al^3+^ and H^+^ and insufficient Ca^2+^ and Mg^2+^ upon root elongation. Eur J Soil Sci. 2003;54(2):323–33.

