# Supplementary figures and images for: Ion Flux in Roots of Chinese Fir (Cunninghamia lanceolata (Lamb.) Hook) under Aluminum Stress
Source: PLoS One. 2016 Jun 6;11(6):e0156832. doi: 10.1371/journal.pone.0156832 (PMC4894599; doi:10.1371/journal.pone.0156832)

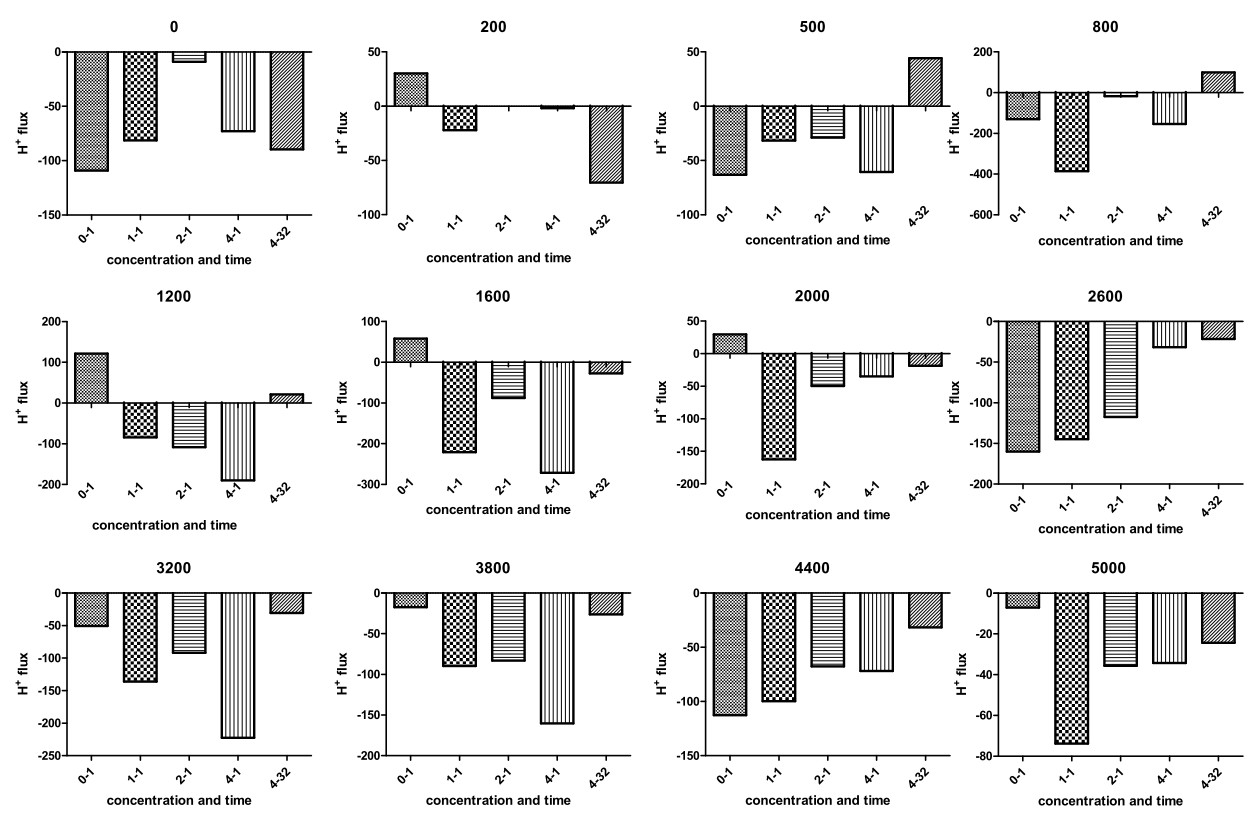

Supplement: S1 Fig — (JPG) [file pone.0156832.s001.jpg]
